# The Association of Nanostructured Carbonated Hydroxyapatite with Denatured Albumin and Platelet-Rich Fibrin: Impacts on Growth Factors Release and Osteoblast Behavior

**DOI:** 10.3390/jfb15010018

**Published:** 2024-01-05

**Authors:** Renata de Lima Barbosa, Neilane Rodrigues Santiago Rocha, Emanuelle Stellet Lourenço, Victor Hugo de Souza Lima, Elena Mavropoulos, Rafael Coutinho Mello-Machado, Carolina Spiegel, Carlos Fernando Mourão, Gutemberg Gomes Alves

**Affiliations:** 1 Graduate Program in Science and Biotechnology, Fluminense Federal University, Niteroi 24210-201, Brazil; 2 Clinical Research Unit, Antonio Pedro Hospital, Fluminense Federal University, Niteroi 24033-900, Brazil; 3 Brazilian Center for Physics Research, Rio de Janeiro 22290-180, Brazil; 4 Department of Implant Dentistry, Universidade Iguaçu, Nova Iguaçu 26260-045, Brazil; 5 Department of Cellular and Molecular Biology, Fluminense Federal University, Niteroi 24033-900, Brazil; 6 Department of Periodontology, Tufts University School of Dental Medicine, Boston, MA 02111, USA

**Keywords:** platelet-rich fibrin, L-PRF, albumin gel, osteoblasts, sticky bone, biomaterials

## Abstract

Platelet-rich Fibrin (PRF), a second-generation blood concentrate, offers a versatile structure for bone regeneration due to its composition of fibrin, growth factors, and cytokines, with adaptations like denatured albumin-enriched with liquid PRF (Alb-PRF), showing potential for enhanced stability and growth factor dynamics. Researchers have also explored the combination of PRF with other biomaterials, aiming to create a three-dimensional framework for enhanced cell recruitment, proliferation, and differentiation in bone repair studies. This study aimed to evaluate a combination of Alb-PRF with nanostructured carbonated hydroxyapatite microspheres (Alb-ncHA-PRF), and how this association affects the release capacity of growth factors and immunomodulatory molecules, and its impact on the behavior of MG63 human osteoblast-like cells. Alb-PRF membranes were prepared and associated with nanocarboapatite (ncHA) microspheres during polymerization. MG63 cells were exposed to eluates of both membranes to assess cell viability, proliferation, mineralization, and alkaline phosphatase (ALP) activity. The ultrastructural analysis has shown that the spheres were shattered, and fragments were incorporated into both the fibrin mesh and the albumin gel of Alb-PRF. Alb-ncHA-PRF presented a reduced release of growth factors and cytokines when compared to Alb-PRF (*p* < 0.05). Alb-ncHA-PRF was able to stimulate osteoblast proliferation and ALP activity at lower levels than those observed by Alb-PRF and was unable to positively affect in vitro mineralization by MG63 cells. These findings indicate that the addition of ncHA spheres reduces the biological activity of Alb-PRF, impairing its initial effects on osteoblast behavior.

## 1. Introduction

In recent decades, several materials and techniques have been developed with the aim of optimizing the bone regeneration process. Blood-based materials have become the target of studies and improvements, giving rise to a range of different blood concentrates featuring a three-dimensional constitution based on fibrin and regenerative potential. The first blood concentrate to gain prominence in the literature was the Platelet Rich Plasma (PRP) [1], obtained from the sequence of centrifuges and the addition of chemical substances, such as sodium citrate. It is presented as a concentrate of platelets and growth factors higher than normal levels, which trigger cascades with different functions in the tissue regeneration environment, such as proliferation, migration, cell differentiation, and angiogenesis, essential in tissue regeneration [2,3,4,5,6].

The second generation of blood concentrates is established by Platelet-rich Fibrin (PRF), obtained through a single centrifugation of a blood aliquot and free from the addition of any substance [2,7,8,9]. Its structure is made up of fibrin, large concentrations of growth factors, and cytokines. The benefits of using PRF for bone regeneration include the formation of a very versatile structure that provides a framework for the population of cells recruited to the site of its implantation, in addition to being a continuous reservoir of growth factors, such as growth factor platelet derivative (PDGF) and vascular endothelial growth factor (VEGF), activated platelets and cytokines that favor the tissue regeneration process [10,11,12,13,14,15,16,17,18,19]. Miscellaneous adaptations to the protocol for obtaining the PRF were described in the literature with the aim of optimizing the composition biochemistry and stability of the PRF membrane [12,20]. Such changes were made in the parameters of speed, time, angulation of the centrifuge rotor, and three-dimensional structure, as presented in the membrane enriched with denatured albumin (Alb-PRF). In relation to Alb-PRF, the processing by denaturation of a portion of the blood centrifuge and its subsequent inclusion in the fibrin mesh causes relevant three-dimensional changes, increasing its degradation time in vivo (in vivo evaluation of the biocompatibility and biodegradation of a new denatured plasma membrane combined with liquid PRF (Alb-PRF) and modifying the cellular distribution and release dynamics of growth factors and cytokines [11].

In addition to production protocol adaptations, combinations of other biomaterials, such as bone and calcium phosphate, to blood concentrates were proposed in several studies, including the concept of the sticky bone [21,22]. The sticky bone blends the intrinsic regenerative bioactivity of PRF with the structural continuity afforded by synthetic bone substitutes, potentially augmenting surgical handling and localization while promoting osseous regeneration [21]. In vivo, evidence demonstrates that the sticky bone boosts progenitor recruitment, bone matrix deposition, and remodeling compared to solo components [23,24,25,26]. In addition, it enables precise contouring and space maintenance conducive to adequate bone fill and contour by stabilizing granules within surgical sites [27]. This mixed composite also streamlines the operative workflow, reducing user demands without sacrificing quality. Previous research combined the properties of blood concentrates with carbonateapatite (cHA) microspheres in a randomized clinical study [27]. This is a biomaterial with suitable properties to be used as a scaffold for cell migration and colonization and features the replacement of hydroxyl groups by carbonate, which contributes to the establishment of a highly resorbable three-dimensional structure, allowing its replacement with newly regenerated bone [28].

The reabsorption capacity of nanostructured carbonated hydroxyapatite after a few weeks of its implantation in critical defects has been proven by several studies [29,30,31,32], this important characteristic being conferred by the surface topography of the biomaterial for the adhesion and proliferation of osteoblasts [33]. Although the association of platelet concentrates with cHA allows better manipulation of the spheres, there was no indication of the acceleration of bone repair [27]. Considering that several pieces of evidence point to a potential effect of PRF on bone tissue, contributing to its regeneration, it is not clear how its association with a biomaterial could affect the performance of this autologous product and its effects on bone cells.

In this context, the objective of this study was to evaluate the association of the membrane enriched with denatured albumin (Alb-PRF) with nanostructured carbonated hydroxyapatite microspheres and to establish a comparison with the albumin membrane without the spheres. The researchers hypothesized that combining the biomaterial with albumin concentrates would create an effective three-dimensional framework. This framework is expected to enhance cellular response by facilitating cell recruitment, proliferation, and differentiation, owing to the presence of a calcium phosphate-based structure and essential growth factors crucial for bone repair.

The specific objectives of the study were (i) to evaluate the three-dimensional structure of albumin membranes with and without cHA microspheres, (ii) to evaluate the in vitro release capacity of growth factors and immunomodulatory molecules, and (iii) to evaluate cell viability, proliferation, and mineralization of MG63 cells after exposure to membrane eluates.

## 2. Materials and Methods

### 2.1. Ethical Aspects

This project was conducted following the guidelines established by the 1975 international Helsinki agreement after evaluation and approval by the ethics committee of the Hospital Universitário Antônio Pedro, approval number 3.432.068 on 2 July 2019.

### 2.2. Preparation of Nanocarboapatite Microspheres

The nanocarboapatite (cHA) microspheres were synthesized and characterized in the Biomaterial’s Laboratory of the Brazilian Center for Research in Physics (Labiomat, CBPF, Rio de Janeiro, Brazil) by the wet precipitation method at 37 °C, without sintering, thus maintaining the nano-microscopic characteristics. The synthesis and characterization of the spheres were reported in a previous study [28]. The sphere size distribution ranged from 425–600 μm. Its powder contained 6% CO_2_ incorporation by weight, with a calcium/phosphate stoichiometry of 2.070, and a mean nanocrystallite size of 23 ± 9 nm. A BET analysis revealed a pore size ranging from 2 nm to 94 nm, and a specific surface area of 95 m^2^/g.

In this study, the biomaterial was further characterized by scanning electron microscopy (SEM: JEOL FEG 250, JEOL^®^, Tokyo, Japan) to examine the morphology and surface of the spheres. The biomaterial was weighed (0.5 g/vial) on a precision scale (UniblocAUY220, Shimadzu^®^, Kyoto, Japan) and then packaged and sterilized by gamma radiation (Gammacell 220, Nordion^®^, Ottawa, ON, Canada) at 15 kGy/sample—a cobalt-60 irradiator at a dose rate of 19.72 Gy/min for 760 min.

### 2.3. Preparation of Alb-PRF and Alb-ncHA-PRF Membranes

Peripheral blood samples were collected from 10 participants of both sexes, aged between 20 and 46 years. Donors were healthy, with no history of recent use of anticoagulant medications. For collection, additive-free 9 mL tubes (Biocon^®^, Vacutube Seco, São Paulo, Brazil), adapters, and 21G scalps were used. The protocol for Alb-PRF obtention [11] was applied, which briefly consists of a single centrifugation at 700 RCF-max lasting 8 min in a horizontal rotor centrifuge (Bio-PRF, Venice, FL, USA).

After centrifugation, the blood components settled, forming distinct layers depending on their respective densities. Therefore, for the production of activated albumin gel, the first two milliliters of the portion of platelet-poor plasma (PPP) located at the upper end of the tube were collected with a 1 mL syringe (Injex^®^, São Paulo, Brazil). The albumin-rich serum underwent an activation cycle at 75 °C lasting 10 min in the APAG equipment (Silfradent, Santa Sofia, Italy). Soon after the end of the process, the syringes containing the gel were stored at room temperature and protected from light until use.

After the time required for the albumin gel to reach a temperature of approximately 37 °C, it was added to 6-well polystyrene plates. For the production of Alb-PRF membranes, the entire available fraction of the liquid phase of the growth factor concentrate and the buffy coat was collected, added to the wells containing the activated albumin gel, and carefully homogenized.

When preparing the Alb-ncHA-PRF membranes, in addition to the liquid phase of growth factor concentrate and buffy coat, nanocarboapatite microspheres were also added on a proportion of 0.5 g per membrane, or 11 wt%. In both cases, polymerization time varied between 5 and 10 min. Polymerized membranes presented a thickness of approximately 3 mm. After the addition of the spheres, most of them sedimented and were evenly distributed along the polymerized membranes.

### 2.4. Production of the Membrane Eluates

After polymerization, the Alb-PRF and Alb-nCHA-PRF membranes (n = 5 per experimental group) were incubated in 4 mL of DMEM culture medium with 1% antibiotic (penicillin–streptomycin) for 24 h or 7 days. At the end of the experimental times, the conditioned media were collected and stored in a −80 °C freezer.

### 2.5. Scanning Electron Microscopy

In order to evaluate the ultrastructural characteristics of the Alb-PRF and Alb-nCHA-PRF membranes, scanning electron microscopy (SEM) was performed on samples from both groups at experimental times of 24 h to evaluate their initial conformation and at the time of 21 days to ascertain their ultrastructural stability over time. Processing for SEM began by washing the membranes with phosphate-saline buffer (PBS) pH 7.4. Afterwards, the samples were fixed with Karnovsky’s solution (2.5% glutaraldehyde and 4% paraformaldehyde) for 1 h. Sequentially, post-fixation was performed with 1% osmium tetroxide in a 0.2 M sodium cacodylate buffer solution, pH 7.4 in a 1:1 ratio. Soon after, samples from both experimental groups were dehydrated in ethyl alcohol at increasing concentrations ranging from 50% to 100% and hexamethyldisilazane (HMDS). The materials were metallized with gold and observed at 15 kV with a scanning electron microscope (JEOL JSM-6490 LV, JEOL, Tokyo, Japan).

### 2.6. Histological Analysis

Histological slides were produced to evaluate the organization of the platelet concentrates and their interaction with the biomaterial, in addition to the distribution of cells in the membranes. Two samples from each experimental group were fixed with 4% paraformaldehyde 24 h after the end of their polymerization. The samples were embedded in paraffin and cut to a thickness of 7 μm. Subsequently, the histological sections were stained with hematoxylin and eosin (HE) and observed at 20× and 40× magnifications with an optical microscope (Axio Observer.A1, Zeiss, Baden-Württemberg, Germany).

### 2.7. Observation of Viable Cells Inside the Membranes

Aiming to evaluate the viability of mononucleated blood cells trapped inside the membranes, a Live/Dead assay was performed. The membranes were maintained in cultures for 7 days in 6-well polystyrene plates, in 4 mL of DMEM culture medium, without FBS and 1% antibiotics (penicillin + streptomycin), with regular changes every 7 days at 37 °C and 5% CO_2_. A commercial cell imaging kit LIVE/DEAD^®^ (Invitrogen, Thermo Fisher Scientific, St. Louis, MA, USA), according to the manufacturer’s directions was employed, whose staining is based on the permeability of cytoplasmic membranes. Viable cells are stained in green by Fluorescein isothiocyanate (FITC), and non-viable cells are stained in red (Texas Red, Thermo Fisher Scientific, St. Louis, MA, USA), with fluorescence in the wavelength ranges of 488 nm/515 nm and 570 nm/602 nm, respectively. Images were collected in a fluorescence microscope (Axio Observer.A1, Zeiss, Germany). The quantification was performed by counting red or green cells in 5 independent fields obtained at 40× magnification, with the help of the Image Pro Plus 6.0 software (Media Cybernetics, Silver Spring, MD, USA).

### 2.8. Quantification of Cytokines and Growth Factor Release

The concentrations of cytokines and growth factors released by the membranes were measured through the analysis of conditioned media at a concentration of 100%. For the detection of biomolecules, a multiparametric immunoassay based on magnetic microbeads labeled with XMap technology (LuminexCorp, Austin, TX, USA) was used, using a commercial kit (27-plex panel, Biorad Inc., Hercules, CA, USA) capable of quantifying IL-1β, IL-1RA, IL-2, IL-4, IL-5, IL-6, IL-7, IL-8 IL-9, IL-10, IL-12 (p70), IL-13, IL-15, IL-17, CCL11, FGF-b, CSF3, CSF2, IFN-γ, CXCL10, CCL2, CCL3, CCL-4, PDGF, CCL5, TNFα, and VEGF. Quantification of magnetic beads and dosages was performed with a Bio-Plex MAGPIX system (Biorad Inc., Hercules, CA, USA). Results were analyzed using Xponent v. 3.0 software (LuminexCorp, Austin, TX, USA).

### 2.9. Culture of Human Osteoblasts

The human osteosarcoma-derived MG-63 cell line was chosen in this study, due to its similarity with normal osteoblasts. The cells were obtained from the Rio de Janeiro Cell Bank (BCRJ) at the #104 passage and cultivated in DMEM (Dulbecco’s Modified Eagle’s Medium), supplemented with 10% fetal bovine serum (FBS) and 1% antibiotic (streptomycin/penicillin). Cultures were maintained in an incubator at 37 °C and an atmosphere with 5% CO_2_. Total changes in the culture medium were carried out at regular intervals of 72 h, and subcultures occurred in a 1:4 ratio.

### 2.10. Evaluation of Cell Viability and Proliferation

MG63 cells were subcultured for 24 h on 96-well plates at a density of 10,000 cells/well (n = 5 per experimental group), and then exposed for 24 h to 5 concentrations of the Alb-PRF and Alb-ncAH-PRF eluates, diluted at 100%, 75%, 50%, 25% or 12.5% in DMEM. After the exposure, the cell viability was determined through the XTT (2,3-bis-(2-methoxy-4-nitro-5-sulphenyl)—(2H)—(tetrazolium-5-carboxanilide) assay by using a commercial kit (In Cytotox, Xenometrics, Allschwil, Germany). A group of cells were exposed to unconditioned media (n = 5) as the experimental control. The optical density (O.D.) at 480 nm was measured with a Sinergy II microplate reader (Biotek Inst., Winooski, VT, USA).

In order to evaluate the effects of the eluate on cell proliferation, MG-63 cells were seeded in 96-well plates at a density of 1000 cells/well and treated with 150 µ of culture medium conditioned with either 25% or 50% of the eluate from the membranes of both experimental groups. The experiment was carried out in quintuplicates, with each replicate treated with membrane eluate from a different donor. The conditioned medium was renewed every 72 h, maintaining the original eluate concentrations. A Crystal Violet Dye Exclusion assay (In Cytotox, Xenometrix, Allschwil, Germany) was performed to estimate cell density relative to the control group (cells exposed to unconditioned media), at 24 h, 3, 5, and 7 days of treatment, by the measurement of the optical density at 540 nm with a Sinergy II microplate reader (Biotek Inst., Winooski, VT, USA).

### 2.11. Determination of In Vitro Mineralization

To compare the osteoinductive potential of the Alb-PRF and Alb-PRF + nCHA membranes, an in vitro biomineralization assay was performed. MG-63 cells at passage 119 were seeded at a density of 20,000 cells/well in 48-well plates and treated with DMEM conditioned with 25% of the membrane eluates, supplemented with 5% FBS and 1% antibiotic (penicillin + streptomycin). Independent experiments were carried out with five technical replicates and five pooled biological replicates, employing cells exposed to unconditioned media as a control group. After the initial treatment, partial changes of the culture medium were carried out every 72 h, equivalent to 50% of the final volume, always keeping the concentrations of the conditioned medium unchanged throughout the entire experimental time.

At 1, 7, 14, and 21 days, the cell supernatant was collected and stored in a −80 °C freezer, and the samples from each group were fixed in 4% paraformaldehyde, and subsequently stained with 40 mM alizarin in an aqueous solution, to stain the deposits of calcium. After extraction with 10% acetic acid, the O.D. at 405 nm was read using a Sinergy II microplate reader (Biotek Inst., Winooski, VT, USA).

The collected cell supernatants were used to determine the alkaline phosphatase activity, using a p-nitrofenilphosphate (PNPP)-based commercial kit (Labtest, Minas Gerais, Brazil), following the manufacturer’s guidelines. To determine the enzyme activity by the hydrolysis of PNPP as a function of time, the optical density at 405 nm was monitored for 2 h in kinetic mode on a Sinergy II microplate reader (Biotek Inst., Winooski, VT, USA). Three independent experiments were performed in quintuplicates.

### 2.12. Statistical Analysis

Comparisons were performed between groups at different eluate concentrations and experimental times. The normality was assessed through the Shapiro–Wilk test and non-parametric analysis of variance was carried out using Kruskal–Wallis with Dunn post hoc test, considering an alpha error of 5%. The statistical analysis was performed using Graphpad Prism 8 software (Graphpad Inc., San Diego, CA, USA).

## 3. Results

**Figure 1:**
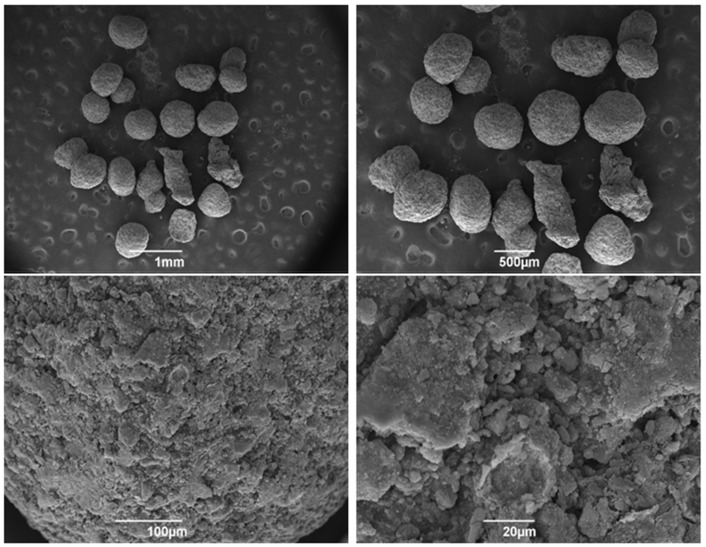
Scanning electron microscopy (SEM) micrographs of nanostructured carbonated hydroxyapatite microspheres. Observe the morphology starting from 1 mm to 20 µm from the surface of the spheres, and they are irregular and full of indentations.

Soon after the synthesis, the ultrastructure of the carbonated hydroxyapatite spheres was evaluated, as shown in Figure 1. At higher magnification, it was possible to observe the roughness of the surface of the spheres, providing an increased surface area.

**Figure 2:**
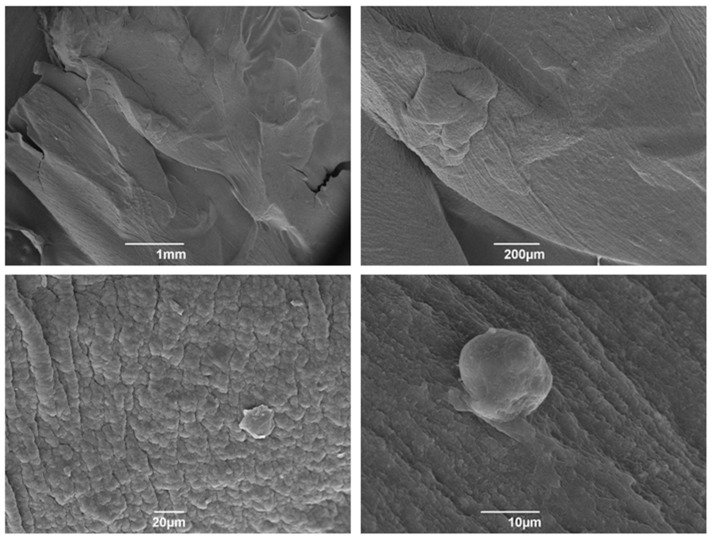
Scanning electron microscopy (SEM) micrographs of 24 h Alb-PRF membranes. The morphology starting from 1 mm to 10 µm indicates a very dense denatured albumin layer surrounding the fibrils of the membrane.

The images obtained 24 h after the synthesis of the Alb-PRF membranes demonstrate the structural robustness of the membrane, substantially different from other types of platelet concentrates (Figure 2).

**Figure 3:**
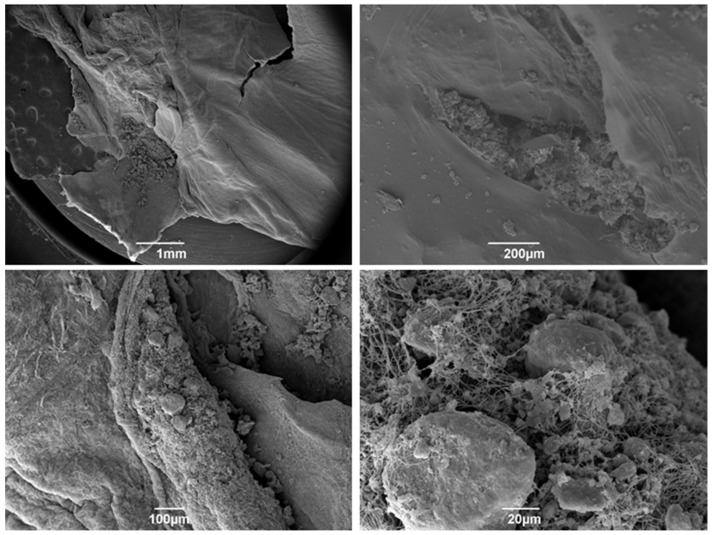
Scanning electron microscopy (SEM) micrographs of PRF albumin membranes associated with 24 h nanostructured carbonated hydroxyapatite microspheres. Observe the morphology starting from 1 mm to 20 µm from the membrane with cells wrapped in the fibrin network.

One day after integrating the spheres on the autologous material, producing the Alb-ncHA-PRF membranes, it is possible to observe the dense appearance resulting from the presence of denatured albumin (Figure 3), and a notable interaction between the fibrin network and the carbonated hydroxyapatite spheres.

**Figure 4:**
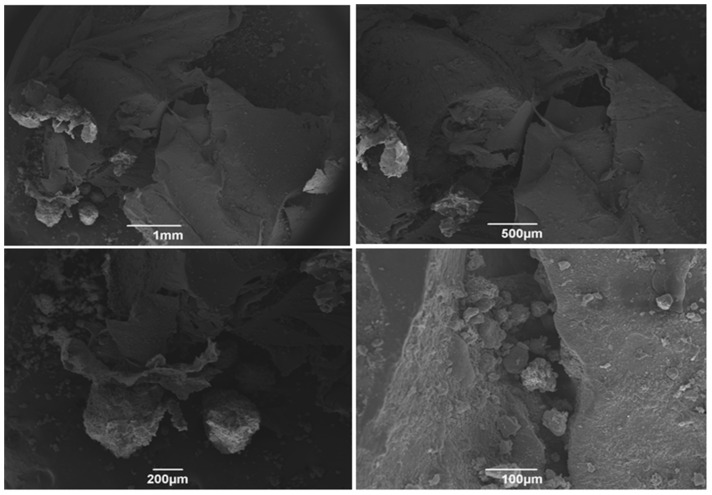
Scanning electron microscopy (SEM) micrographs of Alb-PRF membranes associated with 21-day nanostructured carbonated hydroxyapatite microspheres. Note the structured denatured albumin layer associated with the remaining carbonated hydroxyapatite microspheres.

After 21 of the synthesis of the Alb-ncHA-PRF membranes, the micrographs show that the initial characteristics of the fibrin–albumin scaffold were maintained (Figure 4). Although slightly degraded, the carbonated hydroxyapatite spheres continue to adhere to the membrane, and an evident interaction with the fibrin network remains observable.

**Figure 5:**
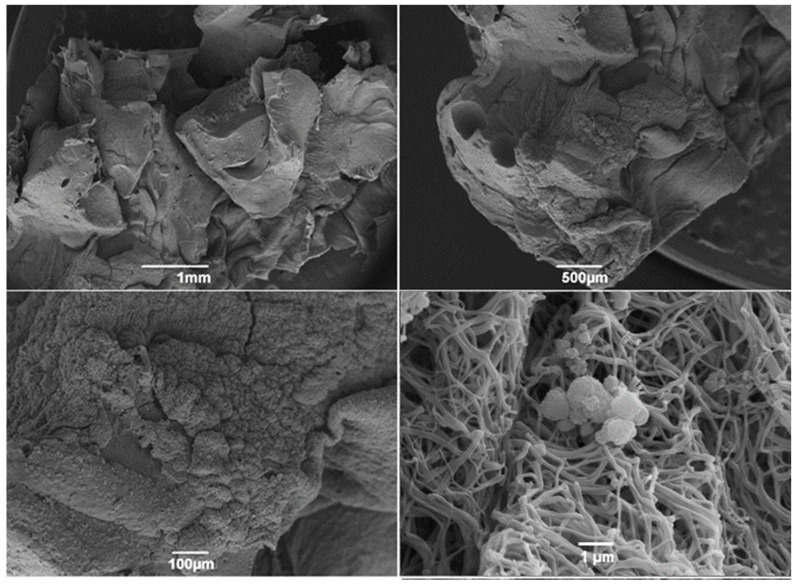
Scanning electron microscopy (SEM) micrographs of 21-day Alb-PRF membranes. Observe the morphology starting from 1 mm to 1 µm of the membrane, indicating a membrane that the dense layer of denatured albumin remains structured 21 days after polymerization, and portions with an intact fibrin network are still visible (lower right panel).

The micrographs of the Alb-PRF membranes at the experimental time of 21 days point to the integrity of the membrane over time (Figure 5).

**Figure 6:**
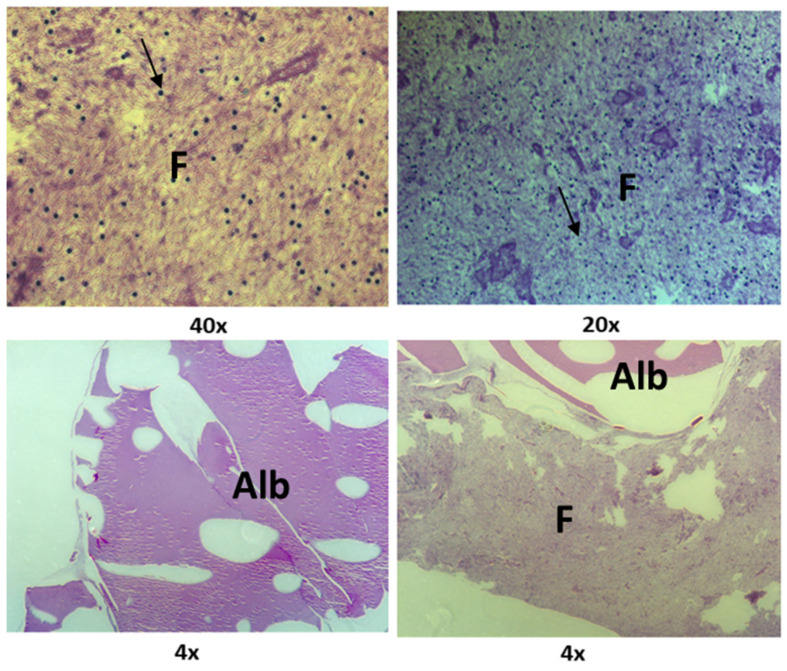
Histological sections of the Alb-PRF membranes. Note that the concentration of cells (arrows) on the fibrin portion (F), in comparison with a dense denatured albumin portion (Alb).

**Figure 7:**
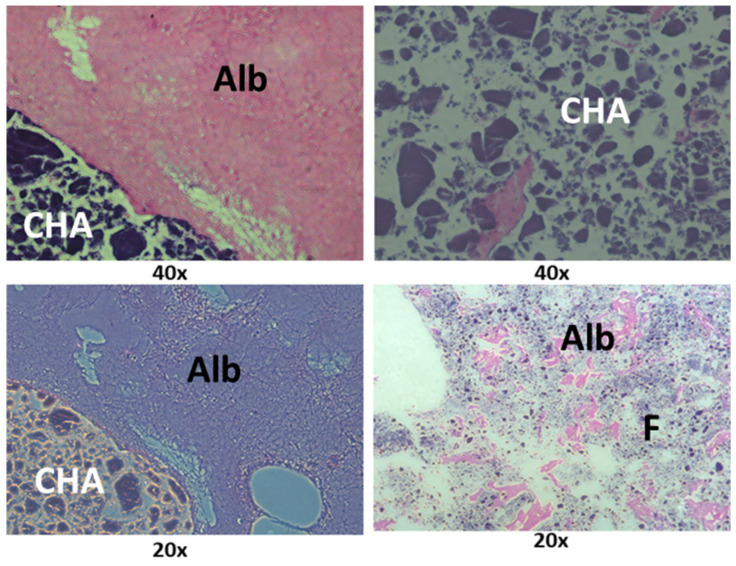
Histological sections of an Alb-PRF membrane associated with nanostructured carbonated hydroxyapatite microspheres (CHA), interacting with both the fibrin (F) and dense albumin (Alb) phases of Alb-PRF.

Figure 6 shows the ultrastructure of the Alb-PRF membranes, evidencing the characteristics of biphasic material, with a portion predominantly fibrous and rich in cells and another dense phase composed of denatured albumin. The addition of nanostructured cHA spheres did not seem to change this pattern, but the spheres were shattered into smaller pieces that often integrated in both albumin and fibrin phases (Figure 7).

**Figure 8:**
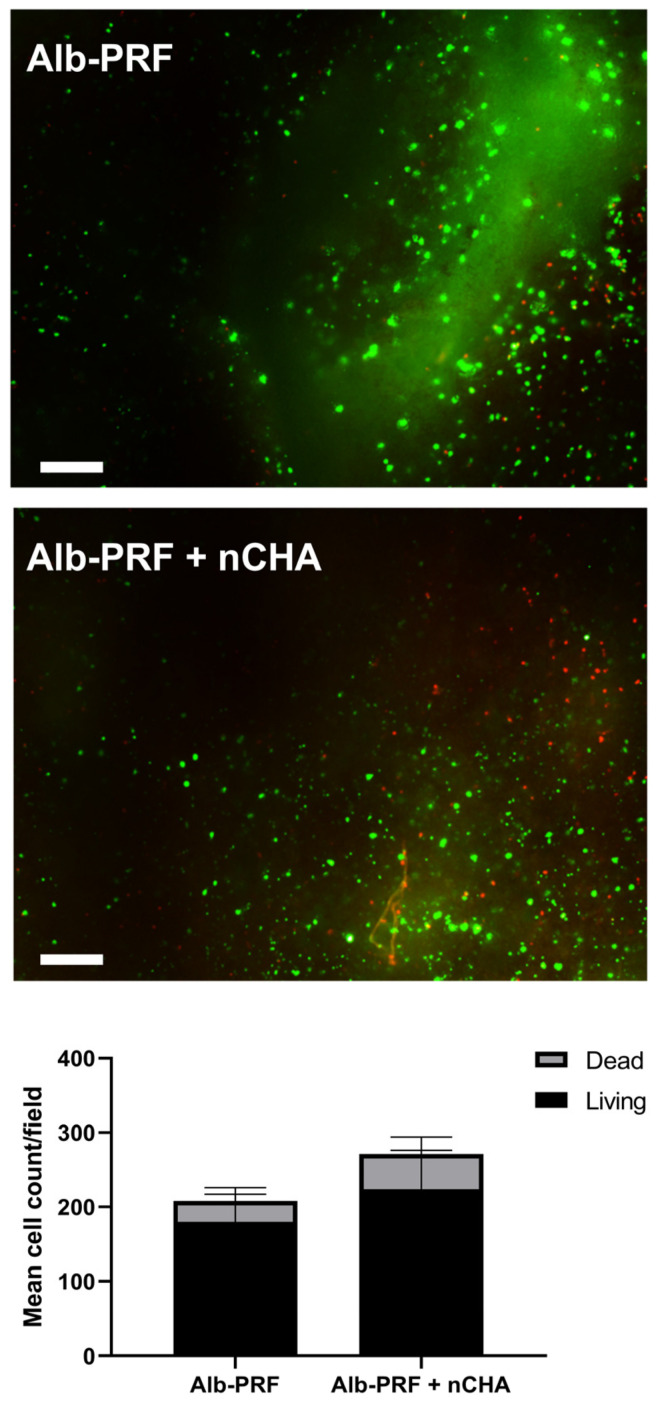
Fluorescence Microscopy of the Live/Dead assay of blood cells present in the Alb-PRF and Alb-PRF + nCHA membranes after 24 h. Green (calcein-AM) staining for live cells, and red (EthD) for dead cells. The scale bar indicates 500 μm. The lower panel shows the mean count ± SD of live and dead cells from 5 independent fields.

A Live/Dead assay was carried out either one or seven days after the production of the membranes to monitor the presence of living blood cells in the Alb-PRF and Alb-ncHA-PRF membranes over time (Figure 8). Both membranes were characterized by abundant calcein staining, indicating the presence of viable cells. The proportion of live cells was 72 and 80% for Alb-PRF and Alb-PRF + nCHA, respectively, without significant difference (*p* > 0.05).

**Figure 9:**
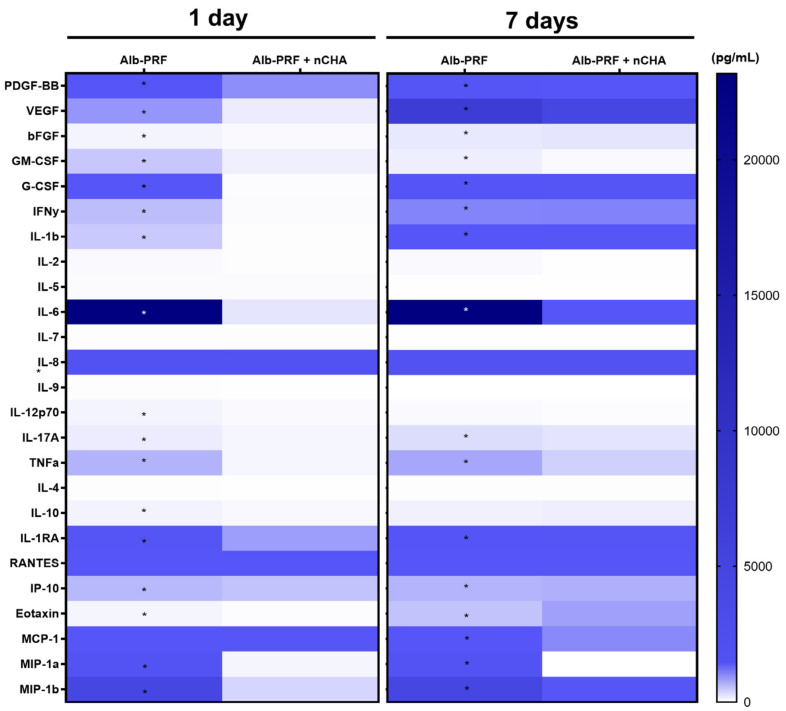
Heatmap of the release of cytokines and growth factors from Alb-ncHA-PRF and Alb-PRF membranes after either 24 h or 7 days of elution in a culture medium. An asterisk indicates a significant difference between groups (*p* < 0.05).

The release of cytokines, chemokines, and growth factors by the Alb-PRF and Alb-ncHA-PRF membranes was assessed in culture media within 24 h. While both membranes were able to release biological mediators (Figure 9), 68% of the 25 assessed molecules were reduced in Alb-ncHA-PRF membranes when compared to Alb-PRF membranes (*p* < 0.05), including all growth factors investigated (PDGF, VEGF, FGF2, GM-CSF, G-CSF). After 7 days of elution, the cumulative release of growth factors by the Alb-PRF + nCHA membranes increased consistently, while remaining significantly lower than Alb-PRF membranes.

**Figure 10:**
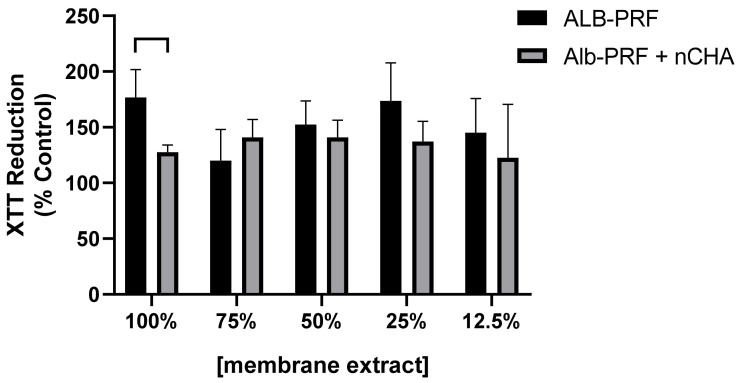
Cytocompatibility assessment of ALB-PRF and ALB-PRF + nCHA membrane extracts, in different dilutions in culture medium (DMEM), measured in MG63 cells, after 24 h of exposure, using the XTT test. The bars show the mean and standard deviation of 3 independent experiments, in quintuplicates, represented as a percentage of the control group (cells exposed to culture medium). The line/lack indicates a significant difference between groups (*p* < 0.05). A positive cytotoxicity control was produced with latex extracts, showing 20.7% average survival.

The cytocompatibility of Alb-PRF and Alb-ncHA-PRF membranes with human bone cells was investigated through an XTT assay, carried out 24 h after treatment of MG63 cells with different concentrations of membrane eluates. Both the Alb-PRF and Alb-ncHA-PRF membranes were biocompatible with cells at all concentrations tested (Figure 10), as they induced similar viability to the unexposed control group (*p* > 0.05). As an exception, when exposed to 100% eluates of Alb-PRF, cells showed significantly higher levels of dehydrogenase/mitochondrial activity compared to Alb-ncHA-PRF and control (*p* < 0.05).

**Figure 11:**
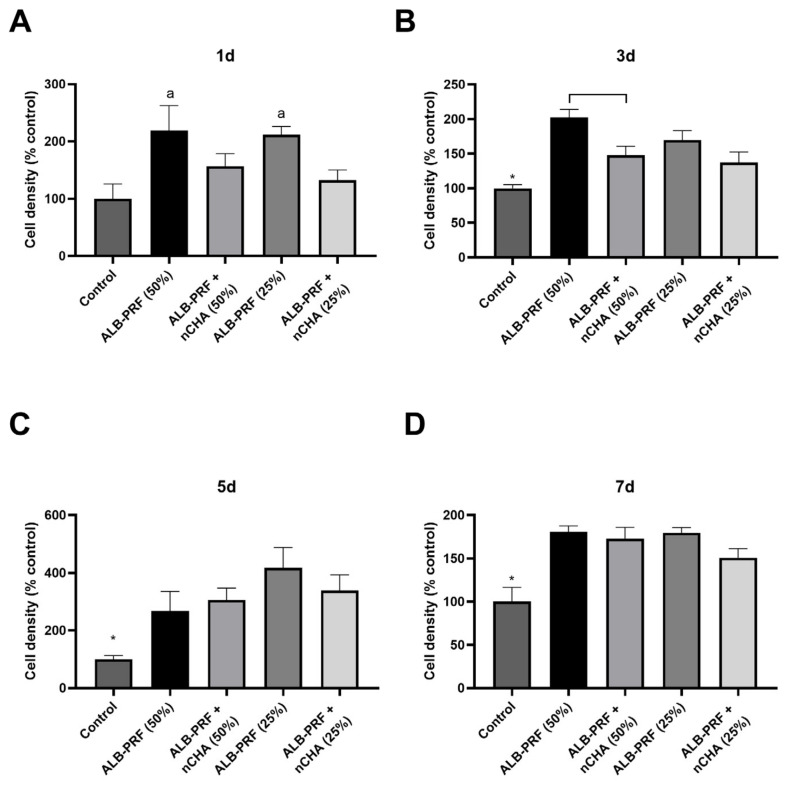
Proliferation of MG63 cells for up to 7 days ((**A**) Day 1, (**B**) Day 3, (**C**) Day 5, and (**D**) day 7) after exposure to Alb-PRF, Alb-nCHA-PRF extracts, expressed as a percentage of the control group (culture medium). Cell density was assessed through the crystal violet dye exclusion test. The letter (a) indicates a significant difference in relation to the control (*p* < 0.05). The bars indicate mean ± standard deviation (n = 5). Asterisks indicate a significant difference (*p* < 0.05) in relation to all other experimental groups. A line indicates a significant difference between groups.

The effects of Alb-PRF and Alb-nCHA-PRF membranes on bone cell proliferation were estimated in samples treated with conditioned media diluted at 25% or 50%. On day 1, only the Alb-PRF membranes significantly stimulated proliferation, without a difference in performance between concentrations of 25% or 50%. On day 3, all experimental groups were effective in stimulating proliferation when compared to the control, but Alb-PRF membranes at a concentration of 50% were more effective than Alb-ncHA-PRF at the same concentration (Figure 11). On the remaining days, both membranes continued to perform better than the control, but without significant difference between them or between the different concentrations used.

**Figure 12:**
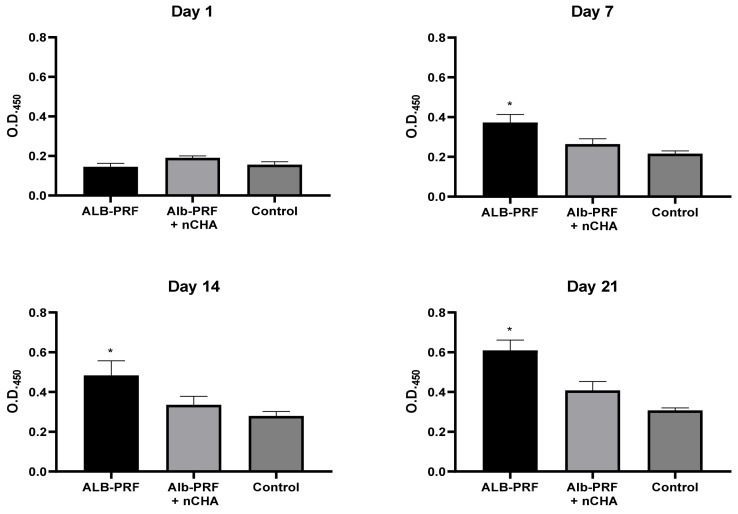
In vitro calcification by MG63 cells exposed for up to 3 weeks to ALB-PRF or ALB-PRF + nCHA eluates. Bars indicate mean ± standard deviation (n = 3) of alizarin-S labeling, expressed by optical density at 450 nm. An asterisk indicates a significant difference (*p* < 0.05) from the control group, exposed to culture medium.

The potential of the Alb-PRF and Alb-PRF + nCHA - membranes to induce in vitro mineralization was evaluated by labeling extracellular calcium deposits with Alizarin Red. One day after exposure to 25% eluates, there was no variation between the experimental groups and the control. From day 7 and throughout days 14 and 21, it is possible to notice a consistent and progressive increase in mineralization induced by the Alb-PRF membrane (*p* < 0.05) (Figure 12). The Alb-nCHA-PRF membrane does not show signs of significantly favoring calcification (when compared to control) at any of the experimental times.

**Figure 13:**
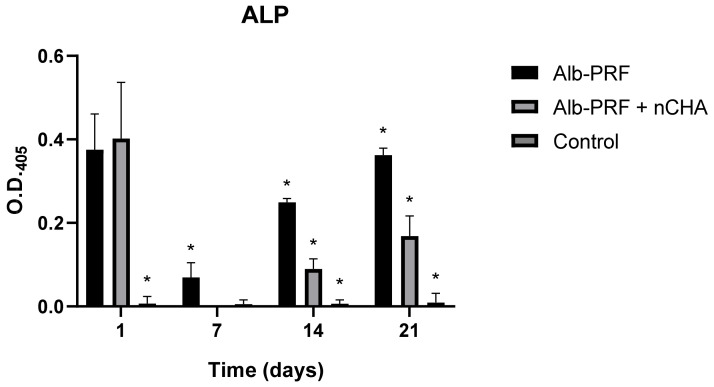
Assessment of alkaline phosphatase enzyme activity in the culture medium over 3 weeks by MG63 osteoblasts exposed to extracts (25%) of ALB-PRF and ALB-PRF + nCHA membranes, or DMEM culture medium (control). The bars show the mean and standard deviation of 3 independent experiments, in quintuplicate, represented as the optical density of the medium, relative to the breakdown of the PNPP substrate. Asterisks indicate significant difference compared to all other groups at the same experimental time (*p* < 0.05).

The alkaline phosphatase (ALP) enzyme activity was estimated in culture media over the three weeks of exposure to the eluates. At 24 h, both the Alb-PRF and Alb-nCHA-PRF membranes showed the highest peaks of enzymatic activity, more than seventy times greater than the control group. From day 7 to day 21, cells stimulated with the Alb-PRF membrane showed increasing enzyme activity, although lower than the peak observed on day 1. The trend of progressive increase in enzyme activity was also seen on days 14 and 21 in cells treated with the Alb-ncHA-PRF membrane eluate when compared to the control, but significantly lower than Alb-PRF (*p* < 0.05) (Figure 13).

## 4. Discussion

The present study aimed to contribute to the development of novel biomaterials by testing the hypothesis that the association of a nanostructured calcium phosphate allograft with Alb-PRF, in addition to representing a potential three-dimensional biodegradable barrier, would improve the behavior of bone cells, since the material provides both calcium phosphate and growth factors, all of which are important aspects for bone repair. The formulation of such a hypothesis was based on three key premises: (i) Alb-PRF exhibits interesting properties as a biological barrier containing bioactive molecules; (ii) nanocaboapatite microspheres have been supported by evidence in the literature for their effects in providing calcium phosphate, enhancing biomineralization; and (iii) the combinations of calcium phosphates with platelet concentrates, recognized as ‘sticky bone’, yields favorable clinical outcomes. Unexpectedly, the results highlighted a limitation attributed to the high protein absorptivity of nCHA, potentially modifying the initial biological response of the resulting composite.

Due to its osteoconductive and biocompatible properties, hydroxyapatite (HA) is widely used as a bioceramic for bone replacement applications. The incorporation of carbonate into nanostructured HA causes significant changes in its physicochemical properties, resulting in increased in vivo dissolution rates and enabling the production of bone substitutes with greater resorption [34,35]. In this study, the ultrastructure of nCHA spheres was evaluated by scanning electron microscopy (SEM), enabling to observe a highly porous surface topography. The porosity of carbonated nanostructured hydroxyapatite spheres is an important parameter that affects their performance as biomaterials for bone tissue engineering. It can be controlled by varying the synthesis parameters, such as temperature, time, pH, and concentration of the reactants, and usually ranges from 20% to 60% [31]. The porosity of nanostructured cHA spheres affects their mechanical properties, biodegradability, bioactivity, and surface area, usually resulting in increased protein adsorption from biological media—a key factor that influences the cellular response and tissue integration of biomaterials [27,32]. According to the work of Anjos et al. (2019) [32], nutrients are consistently adsorbed from biological media by nanostructured nanohydroxyapatite, however, without impact on cell growth or survival on the in vitro assessments performed by the authors.

The membranes produced by the Alb-PRF protocol were observed in detail by scanning electron microscopy and histology, identifying a very similar pattern from previous reports of the fibrin membrane enriched with denatured albumin [11,36,37], exhibiting a dense surface, in which the deposition of a layer of denatured protein was evident that entirely surrounded the fibrin fibers, as well as the retention of leucocytes and platelets. This structural configuration can be considered advantageous for guided bone regeneration (GBR) purposes. Guided bone regeneration (GBR) represents a surgical approach designed to repair lost bone and soft tissue encircling dental implants or in areas affected by periodontal issues. GBR demands the utilization of biological barriers intended to block the infiltration of undesired cells into the affected area, allowing the growth of osteogenic cells inside the defect area. The most common barriers are non-resorbable, based on polytetrafluoroethylene (PTFE), while resorbable barriers eliminate the need for subsequent surgery for removal [38,39,40,41,42]. An instance of a biological barrier used in GBR is the collagen membrane, produced from bovine or porcine origins that is biocompatible and demonstrates the ability to stimulate bone formation and aid in wound healing. Nonetheless, they come with certain limitations, such as low mechanical strength, rapid breakdown, and potential for immune reactions, and are often enhanced with cross-linking, coatings, or the integration of growth factors or nanoparticles [43,44,45,46]. In this sense, the proposal of Alb-PRF associated with nCHA intended to provide an autologous alternative for a resorbable but stable membrane, maintaining both the release of GFs and hydroxyapatite nanoparticles that could serve, respectively, as inductors and building blocks for bone regeneration.

The addition of nCHA spheres produced membranes with a similar pattern, however, with the presence of smaller shards of biomaterial resulting from the disintegration of the spheres, which could be observed in both phases. It is important to notice, however, that some spheres that interacted with the fibrin portion (wrapped inside fibrils, as observed by SEM) remained relatively integrous for at least 21 days, suggesting that this combination might reduce the solubility of this material and may potentially modulate its solubility. It is an interesting factor to be considered and investigated in future studies, as controlling bioresorption may optimize the bioavailability of calcium and phosphate ions, which are essential for bone mineralization and osteogenesis. Conversely, by decreasing the solubility of CHS, it is possible to prolong their retention time in the bone defect site and provide a sustained release of therapeutic ions [47,48].

The release of cytokines and growth factors by PRF membrane has been widely known since studies in the early 2000s [2,10,13,15,49], resulting in effects on the promotion of angiogenesis, migration, proliferation, and differentiation of mesenchymal cells, fibroblasts, and osteoblasts, among others [50]. In this study, high levels of growth factors such as PDGF, VEGF, and FGF2 were released by Alb-PRF within 24 h. However, with the addition of the nanostructured cHA spheres, the release was significantly reduced for all these molecules, along with several cytokines and chemokines. This finding suggests that the spheres may be trapping different proteins contained within the fibrin scaffold, a feature with great potential to impact the biological effects of PRF during tissue regeneration. Indeed, hydroxyapatite and other calcium phosphates are well known for their ability to adsorb proteins, including growth factors from biological media, such as blood plasma, serum, and cell culture media [51]. While such interactions may be interesting for the controlled delivery of biological mediators in some settings [51,52], the insufficient control over the release profile of GFs may be an issue in the applicability of composite materials such as Alb-ncHA-PRF, as they could cause the loss of expected effects. In this regard, an interesting observation comes from the eluate evaluation at 7 days, where the concentration of GFs released by Alb-PRF + nCHA consistently increased. Even though they remained lower than Alb-PRF alone, it is possible that bioactive molecules are being released as the nCHA spheres slowly dissolve. This result raises the possibility that the biological effects of Alb-PRF associated with CHA may happen at later times after grafting, a hypothesis to be assessed in further studies.

To identify the potential impact of these changes observed in the properties of Alb-PRF caused by the association with ncHA on the behavior of bone cells, we have performed an in vitro assessment employing human osteoblast-like cells derived from osteosarcoma from the cell line MG63. This is a cell type commonly used as an experimental bone cell model due to their similarity to primary human osteoblasts: although there are variations in terms of morphology and shorter duplication time, these cells express the main biological markers of osteoblast behavior, are regulated by the same differentiation regulatory pathways, and can produce a mineralized matrix when adequately stimulated. In this way, they present important characteristics for testing biomaterials in medical applications [53,54].

The cytotoxicity of platelet-rich fibrin (PRF) on osteoblasts is a topic of interest for tissue engineering and regenerative medicine since the optimal concentration of PRF and its cytokines for osteoblast viability and function is still under debate. Silva et al. (2021) [55] investigated the effects of different concentrations of PRF and its cytokines on the cytotoxicity of osteoblasts, reporting that higher doses of PRF elute induced significant cell death and reduced cell viability. These effects were mainly correlated with the concentration of cytokines such as IL-6 and TNF-α. In the present study, Alb-PRF membranes (added or not with cHA) presented good cytocompatibility in all tested concentrations, regardless of the high release of cytokines already identified after 24 h elution. These results are in accordance with previous in vitro assessments of Alb-PRF with other cell types, such as fibroblasts [56,57]. Nevertheless, since the cumulative production and release of cytokines by Alb-PRF membranes have already been reported in the literature [11], we decided to perform the subsequent biological assessments with diluted membrane eluates (50% or 25%) in order to avoid potential cytotoxicity in longer experimental times.

Cell proliferation is a very important phenomenon in tissue regeneration and one of the most positively affected by the growth factors usually released by PRF (such as VEGF, PDGF, and TGF-Beta). The present results confirm that Alb-PRF is able to stimulate the proliferation of human osteoblasts, and its effects at the first 1 to 3 days are significantly stronger than those of Alb-ncHA-PRF. This result provides a first glance at the potential impact of the lower release of biological mediators induced by the addition of ncHA to the platelet concentrates. PDGF, FGF2, and VEGF are important cytokines that contribute to the regulation of the proliferation of osteoblasts, as they bind to specific receptors and activate intracellular signaling pathways that regulate gene expression, cell cycle progression, and survival. PDGF and VEGF also modulate the expression of other factors that influence osteoblast function, such as osteoprotegerin, osteocalcin, and bone morphogenetic proteins [58]. The balance between these growth factors, which was strongly affected by the eluates determines the rate and extent of osteoblast proliferation and differentiation, as well as their interactions with other cells in the bone microenvironment [59].

In vitro mineralization is another of the main parameters investigated to predict the impacts of blood concentrates and PRF derivatives, as it is a marker of the osteogenic potential of these materials and fundamental for bone regeneration. This mineralization involves osteoblast differentiation pathways, such as the cbfa-1/runx2 pathway, activated, in the case of PRF, by growth factors released by the autologous biomaterial [50,60,61,62]. The presence of calcium phosphate (hydroxyapatite) in osteoblastic cell cultures is commonly quantified by the alizarin red staining method, an established approach to characterize a mineralized matrix resulting from osteogenic cell differentiation [63,64]. Through these methods, a significant difference was observed in the calcification induced by Alb-PRF and Alb-ncHA-PRF: while the exposure to Alb-PRF eluates greatly induced in vitro mineralization, this effect was reversed by the presence of ncHA spheres, since the Alb-ncHA-PRF eluates were statistically equivalent to the control group (*p* > 0.05). This is yet another indication that the presence of the spheres affected the biological properties of Alb-PRF, by impairing behaviors that are often modulated by growth factor stimulation.

Alkaline phosphatase (ALP) is widely expressed in mineralized tissue cells and plays a crucial role in the formation of hard tissue, being considered an early marker of osteogenic differentiation [65]. Alkaline phosphatase secretion by osteoblasts increases during early differentiation as cells are preparing for bone matrix synthesis. In the extracellular matrix formation phase, ALP facilitates the mineralization of the newly formed bone matrix. The most significant peak in alkaline phosphatase activity occurs during mineralization and deposition of hydroxyapatite crystals onto the bone matrix, followed by a decrease in ALP levels, reflecting a reduction in active osteoblasts, with some transitioning into osteocytes embedded within the bone matrix or undergoing apoptosis. In this study, ALP activity presented two peaks at days #1 and #21 for both stimulated groups, without relevant ALP secretion by unstimulated cells (control). As recently reviewed by Lima et al. [50], this is one of the most common responses of bone/mineralized tissue cells to stimulation by L-PRF (and its derivative protocols), which was once again, in our study, strongly reduced by the addition of nanostructured cHA spheres into the platelet concentrates scaffold.

Altogether, these findings suggest that the association of denatured albumin-enriched platelet-rich fibrin with nanocarboapatite microspheres decreases the initial expected stimulation of bone cells by the PRF component, most probably through a lower release of cytokines and growth factors. This hypothesis may both be explained and reinforced by the clinical study by Mourão et al. [27], which reported a lack of stimulation of novel bone formation by a “sticky bone” preparation composed of blood concentrate associated with nCHA spheres very similar to those of the present work. It is possible that the difference between this and other studies, reporting a clinical success obtained with associations of PRF and calcium phosphates, lies in a lower protein absorption of proteins due to different physicochemical properties—another hypothesis that still demands confirmation by further studies. Nevertheless, the present outcomes underscore a limitation or a characteristic that material developers should consider when exploring or developing combinations of calcium phosphate and platelet concentrates. Furthermore, these results may contribute to a deeper comprehension of growth factor (GF) release dynamics within this composite material. This understanding presents opportunities for further research aimed at modulating material solubility, consequently influencing the release of biological modulators, as a prospect to effectively regulate and enhance bone tissue regeneration.

## 5. Conclusions

The results indicate that the addition of nanocarboapatite microspheres reduces the initial biological activity of Alb-PRF, associated with a lower release of proteins such as growth factors, which impairs its expected effects on proliferation, in vitro mineralization, and alkaline phosphatase activity of osteoblasts.

## Data Availability

Data are contained within the article.
